# Age-Related Differences in Clinical and Surgical Outcomes Following Oblique Lateral Interbody Fusion: A Retrospective Cohort Study

**DOI:** 10.7150/ijms.127252

**Published:** 2026-08-24

**Authors:** Yun-Che Wu, Cheng-Hung Lee, Jun-Sing Wang, Meng-Jen Lee

**Affiliations:** 1 Department of Orthopedics, Taichung Veterans General Hospital, Taichung, Taiwan.; 2 Department of Post-Baccalaureate Medicine, College of Medicine, National Chung Hsing University, Taichung, Taiwan.; 3 Department of Food Science and Technology, Hung Kuang University, Taichung, Taiwan.; 4 Division of Endocrinology and Metabolism, Department of Internal Medicine, Taichung Veterans General Hospital, Taichung, Taiwan.; 5 Department of Medicine, School of Medicine, National Yang Ming Chiao Tung University, Taipei, Taiwan.; 6 Department of Music, Tunghai University, Taichung, Taiwan.; 7 Department of Applied Chemistry, Chaoyang University of Technology, Taichung, Taiwan.

**Keywords:** oblique lateral interbody fusion, elderly, spine surgery, patient-reported outcomes

## Abstract

**Introduction:**

We compared patient outcomes following oblique lateral interbody fusion (OLIF) for degenerative lumbar spine conditions among different age groups.

**Materials and Methods:**

This retrospective cohort study included patients between January 2018 and July 2020. Patients were stratified into three groups based on age at the time of surgery (<65 years, 65 to <75 years, and ≥ 75 years). Preoperative and postoperative patient-reported outcome measures were assessed, including visual analog scale (VAS) for back and leg pain, Oswestry Disability Index (ODI), and EuroQol-5 Dimension (EQ-5D). Postoperative orthopedic events, including screw loosening, cage subsidence, and revision surgery, were recorded. All patients' survival status were followed until March 2023. Cox-proportional hazard models were conducted to examine the associations of age groups with postoperative orthopedic events and all-cause mortality.

**Results:**

A total of 170 patients were analyzed (n=77, 68, and 25 for age <65 years, 65 to <75 years, and ≥ 75 years, respectively). VAS, ODI, and EQ-5D significantly improved after OLIF in all groups (all p<0.001). There were no significant differences in rates of postoperative screw loosening (p=0.702), cage subsidence (p=0.095), and revision surgery (p=0.265) across the three age groups. After a median follow up of 4.06 years, the three groups had no significant difference in orthopedic event-free survival (log-rank p=0.302). The findings were consistent after multivariate adjustment.

**Conclusion:**

OLIF is a safe and effective surgical option for elderly patients with degenerative lumbar spine conditions.

## Introduction

The global population is aging rapidly, with the number of individuals aged 65 years or older projected to double over the next three decades 1. As longevity increases, so does the prevalence of degenerative spine conditions, including lumbar spinal stenosis, spondylolisthesis, and degenerative disc disease 2,3. These pathologies are leading causes of disability and diminished quality of life among the elderly, often resulting in chronic pain, reduced mobility, and dependence on caregivers 4. Surgical intervention is increasingly considered for elderly patients when conservative treatment fails. However, the risks and benefits of spinal surgery in this population remain a topic of active debate due to concerns about frailty, comorbidities, and potential postoperative complications 5.

Traditional open spinal fusion techniques are associated with significant morbidity, including extensive soft tissue disruption, prolonged operative times, increased blood loss, and extended hospital stays 6. These factors are particularly detrimental to elderly patients, who often have diminished physiological reserves and a higher burden of comorbidities. To address these concerns, minimally invasive surgical techniques have been developed to reduce operative trauma and promote faster recovery. Among these, oblique lateral interbody fusion (OLIF) has emerged as a promising alternative to traditional posterior lumbar fusion, especially in high-risk or elderly populations 7,8.

OLIF offers several potential advantages: reduced muscular dissection, minimized intraoperative bleeding, shorter hospital stays, and quicker rehabilitation 9,10. Additionally, OLIF enables restoration of disc height and sagittal alignment with lower rates of iatrogenic nerve injury compared to direct lateral approaches such as extreme lateral interbody fusion 11,12. Nevertheless, it is unclear whether elderly patients derive similar functional and quality of life improvements from OLIF as younger patients, and whether the risks of surgery remain acceptable in this vulnerable group. In this study, we investigated patient-reported outcome measures (PROMs) and perioperative outcomes in patients underwent OLIF across three age groups (<65 years, 65 to <75 years, and ≥ 75 years).

## Materials and Methods

This retrospective cohort study included patients who underwent OLIF with posterior instrumentation at our institution between January 2018 and July 2020. The study was approved by the institutional review board (approval number CE20218B), and was conducted in accordance with the Declaration of Helsinki. Patient informed consent was waived due to the retrospective study design, and de-identified data were used for analyses. Patients were included if they underwent OLIF for degenerative lumbar spine conditions and had at least one year of clinical follow-up. Exclusion criteria included prior lumbar fusion surgery, trauma, infection, tumor, or incomplete clinical data. Most patients were referred from primary care facilities after unsuccessful initial non-surgical management. Upon referral, patients underwent further medical treatment and/or physical therapy at our institution, during which surgical options were discussed. This evaluation and management period typically lasted approximately one month. There was no age-specific selection criteria. Patients were stratified into three groups based on age at the time of surgery (<65 years, 65 to <75 years, and ≥75 years). Clinical, surgical, and radiographic data were retrospectively reviewed.

Baseline demographic information, comorbidities, and perioperative variables were collected from electronic medical records. Preoperative and postoperative PROMs were assessed, including visual analog scale (VAS) 13 for back and leg pain, Oswestry Disability Index (ODI) 14, and EuroQol-5 Dimension (EQ-5D) 15. A higher VAS and ODI score indicates greater severity of pain and disability, respectively. In contrast, a higher value of EQ-5D indicates a better quality of life. These outcome measures were evaluated preoperatively, and at 1, 3, and 6 months postoperatively. We assessed preoperative bone quality with vertebral bone quality (VBQ) scores calculated using preoperative T1-weighted MRI images of mid-sagittal slice of the lumbar spine according to previously reported methodology 16. To calculate the VBQ score, signal intensity (SI) measurements of the vertebral bodies (L1-L4) were obtained from mid-sagittal T1-weighted MRI sequences. Regions of interest (ROIs) were manually placed at the center of each vertebral body (L1-L4), carefully avoiding the endplates and venous plexuses. A reference ROI was placed within the cerebrospinal fluid at the L3 level to serve as the denominator for the ratio. The VBQ score was calculated by dividing the median SI of the vertebral bodies (L1 to L4) by the SI of the cerebrospinal fluid at the level of the L3 vertebra. The VBQ score ranges from 1 to 5, and a higher score indicates poorer bone quality.

The OLIF procedure was performed through a left-sided retroperitoneal oblique approach between the psoas muscle and the great vessels. After establishing the retroperitoneal corridor, the disc was incised, followed by thorough discectomy and endplate preparation under fluoroscopic guidance. An appropriately sized interbody cage filled with graft material was then inserted obliquely to achieve disc height restoration and indirect neural decompression. Posterior fixation was subsequently performed using either percutaneous pedicle screw placement or a more extensive open posterior approach. Because indirect decompression often yields an inadequate response in patients with frank osteoporosis or severe spinal stenosis—typically resulting in less than a 50% reduction in leg and back pain at rest—direct decompression 17 was usually performed in patients with facet fusion on CT, free disc fragments, or compressive facet joint cysts.

Surgical parameters, including number of fusion levels, operative time, intraoperative blood loss, and length of hospital stay, as well as postoperative orthopedic events, such as screw loosening, cage subsidence, and revision surgery, were recorded. Operative time was defined as the skin-to-skin time, encompassing the entire procedure from the initial OLIF incision to the final posterior closure. Screw loosening was defined as the presence of a circumferential radiolucent zone (the “halo sign”) greater than 1 mm in width around the pedicle screw on anteroposterior or lateral radiographs. We assessed cage subsidence on lateral radiographs using the Marchi criteria 18, which classify the extent of cage migration into the vertebral endplates based on the percentage of disc height loss relative to the immediate postoperative state. Cage subsidence was defined as a loss of more than 50% of disc height. Interbody fusion was assessed according to the Bridwell grading system, where Grades I-II denote solid or probable fusion, and Grades III-IV correspond to probable or definite non-union. The postoperative orthopedic events were assessed using radiographs obtained postoperatively and at 3, 6, and 12 months after OLIF. For all patients, CT scans at 12 months were used to correlate with radiographic findings and to confirm the Bridwell fusion grade. Revision surgery was confirmed based on operative records. All patients' survival status was followed until March 2023.

### Statistical analysis

Continuous variables are presented as mean ± SD, and categorical variables are expressed as frequencies and percentages. VBQ scores were measured by two blinded raters. Before measuring the study cases, interobserver reliability was assessed using the intraclass correlation coefficient (ICC) in 20 randomly selected cases. The interobserver ICC for VBQ measurement was 0.93 (95% CI, 0.85-0.97). Comparisons among the three age groups were conducted using the one-way analysis of variance (one-way ANOVA) for continuous variables and the chi-square test for categorical variables. Repeated measures within groups across time points were analyzed using paired t-tests. To account for within-subject correlation and to allow inclusion of participants with incomplete follow-up, repeated PROM measurements were analyzed using linear mixed-effects models adjusted for age and sex. Missing data were handled using multiple imputation under the missing at random assumption. Kaplan-Meier survival curves were plotted for the three age groups (<65 years, 65 to <75 years, and ≥ 75 years). Cox-proportional hazard models were conducted to examine the associations of age groups with postoperative orthopedic events and all-cause mortality with adjustments for sex, concomitant chronic diseases, and preoperative bone quality (VBQ). We tested the proportional hazards assumption for Cox models and the assumption was satisfied (p=0.256). A p-value of less than 0.05 was considered statistically significant. All statistical analyses were performed using SPSS software (version 22.0, IBM Corp., Armonk, NY, USA).

## Results

A total of 170 patients who underwent OLIF were included in this study. Patients were divided into three groups according to their age: <65 years (n=77), 65 to <75 years (n=68), and ≥ 75 years (n=25). The baseline characteristics of the study patients are summarized in Table 1. The mean age of the three groups were 56.1 ± 7.3, 68.9 ± 2.6, and 77.0 ± 2.3 years (p<0.001), respectively. Patients with age ≥ 75 years were more likely to have diabetes (52.0%) and hypertension (80.0%), and had poorer bone quality (higher VBQ) and higher ODI (61.3 ± 7.1) than the younger groups (all p<0.05). There were no significant differences in the other variables among the three groups (Table 1).

Table 2 shows changes in PROMs after OLIF across different age groups. The numbers of patients with missing PROM data at baseline, 1, 3, and 6 months were 3 (1.8%), 11 (6.5%), 17 (10.0%), and 31 (18.2%), respectively. VAS significantly improved at 1-month, 3-month, and 6-month follow-up (all p<0.001 vs. baseline) without significant difference among the three groups. Similarly, ODI significantly decreased and EQ-5D significantly improved after OLIF in all the three groups. Nevertheless, patients with age ≥ 75 years had higher ODI (at all time points) and lower EQ-5D (at 3-month and 6-month follow-up), as well as smaller improvements in these indices, than the younger groups.

Linear mixed-effects models were used to assess changes in repeated measurements of PROMs (Table 3). Decreases in VAS and ODI scores and improvements in EQ-5D were observed at all follow-up time points (all p<0.001). There were no significant differences in changes in VAS across the three age groups. In contrast, the reduction in ODI was smaller in the elderly groups than in patients aged <65 years. A similar pattern was observed for EQ-5D. The interaction between time and age group was examined in the model, yielding p values of 0.234, <0.001, and <0.001 for VAS, ODI, and EQ-5D, respectively (Table 3).

Surgical details and postoperative complications are presented in Table 4. The average number of surgical levels were similar among the groups (2.3 ± 1.6 vs. 2.2 ± 1.4 vs. 2.2 ± 1.5, p=0.882). The operation time was modestly shorter in patients with age ≥ 75 years (p=0.012). There was no significant difference in blood loss (p=0.085) and length of hospital stay (p=0.128) among the three groups. With regard to adverse post-operative outcomes, the differences in rates of screw loosening (p=0.702), cage subsidence (p=0.095), revision surgery (p=0.265), and fusion (p=0.123) were not significant across the age groups (Table 4).

Figure 1 shows the survival curves of adverse orthopedic event-free (screw loosening, cage subsidence, and revision surgery) survival of the three age groups during a median follow up of 4.06 years. There was no significant difference with regard to orthopedic event-free survival (log-rank p=0.302) across the three groups. We examined the associations of age groups with any orthopedic event and all-cause mortality in Table 5. Using patients with age <65 years as the reference group, the other two groups were not associated with statistically significant risk of post-operative orthopedic events and all-cause mortality after multivariate adjustment.

## Discussion

This study evaluated PROM and perioperative outcomes in patients underwent OLIF across three age groups. Our findings revealed that elderly patients achieved significant improvements in pain relief, disability scores, and quality of life over a 6-month period, while older patients showed smaller improvements in ODI and EQ-5D and had worse absolute postoperative outcomes (Table 2 and Table 3). Importantly, there were no significant differences in the complication rates—including screw loosening, cage subsidence, and revision surgery—across the three age groups, suggesting that OLIF can be a safe and effective option even in older adults. Our results align with growing evidence that minimally invasive fusion techniques, including OLIF, can yield favorable outcomes in elderly populations 19,20. Several studies have highlighted the reduced surgical morbidity associated with OLIF compared to traditional posterior approaches, which is particularly beneficial for elderly individuals who often have limited physiological reserves 21,22. Despite having significantly higher rates of diabetes and hypertension, as well as ODI, our patients with age ≥ 75 years did not experience increased postoperative complication rates compared with the younger groups. This suggests that careful patient selection and meticulous perioperative management can mitigate age-related surgical risks 23.

Significant improvements were observed in VAS, ODI, and EQ-5D scores postoperatively across all age groups, demonstrating the effectiveness of OLIF in alleviating pain and improving function. However, the extent of improvement was less pronounced in the elderly group. For instance, while patients with age <65 years had a 26.8-point reduction in ODI by 6 months (from 52.2 ± 8.8 to 25.4 ± 15.9), those with age ≥ 75 years showed a 13.1-point decrease (from 61.3 ± 7.1 to 48.2 ± 7.0) (Table 2). The findings are consistent with previous literature indicating that older adults, although capable of functional gains post-surgery, may experience diminished improvements due to preexisting frailty, sarcopenia, and less physiological plasticity 24,25. Furthermore, the smaller gain in EQ-5D scores among elderly patients reflects a more limited enhancement in perceived quality of life. This could be attributed to baseline health status, which may influence patients' subjective evaluation of their postoperative recovery 26. Prior studies have found similar trends, where patient-reported outcomes plateau at a lower level in the elderly, even after technically successful surgeries 27. Nevertheless, the clinical significance of these gains should not be underestimated. In our study, elderly patients still experienced statistically and clinically meaningful improvements, confirming that age alone should not be a contraindication for OLIF. Rather, it should prompt individualized risk-benefit assessments that consider comorbid conditions, functional reserve, and patient goals 28.

Interestingly, older patients in our study had significantly shorter operative times and less intraoperative blood loss than their younger counterparts. Preoperative bone quality (VBQ) was even poorer in the elderly group (Table 1). Surgeons might adopt more conservative strategies in elderly patients, such as minimizing the number of fused levels or avoiding complex decompressive procedures when indirect decompression may be sufficient. Unfortunately, we did not observe differences in the number of fusion levels, complexity of decompressions, or other surgical factors that could explain this finding. Experienced surgeons may complete procedures more efficiently in older adults to reduce surgical stress and anesthesia time. This finding is corroborated by Wang et al. 29, who reported shorter operative durations and reduced bleeding in older adults undergoing minimally invasive spine surgery, possibly due to more straightforward surgical plans tailored to reduce risks. These factors may explain why our elderly group did not experience significantly longer hospital stays (9.6 ± 4.4 vs. 7.8 ± 4.7 days in those with age <65 years, Table 4) despite higher comorbidity burdens. Extended hospital stays in older adults have been attributed to slower mobilization, greater need for post-acute rehabilitation, and careful monitoring for medical complications 30. Previous literature has noted that even when minimally invasive techniques are used, discharge readiness is often delayed in older patients due to lower preoperative function and postoperative fatigue 31. While OLIF facilitates early mobilization and functional recovery, age-related factors may still influence the overall pace of postoperative rehabilitation. Enhanced recovery protocols specifically adapted to geriatric patients undergoing spine surgery may help bridge this gap 32,33.

One of the most reassuring findings of our study is that rates of screw loosening, cage subsidence, and revision surgery did not significantly differ among the three age groups. This observation reinforces the safety of OLIF in elderly patients and echoes findings from recent studies indicating low complication rates in older adults undergoing minimally invasive surgical fusion 19,20. Screw loosening and cage subsidence are of particular concern in the elderly due to osteoporosis. However, our study did not find a higher incidence in the elderly group, possibly due to techniques aimed at maximizing fixation in osteoporotic bone, such as longer screw purchase, cement augmentation, and use of expandable cages. Moreover, elderly patients may be more likely to undergo preoperative evaluation for bone quality, allowing for appropriate planning and device selection. This is in line with previous study that preoperative bone health assessment is important to reduce implant-related complications 19.

As expected, the prevalence of diabetes and hypertension was significantly higher in the elderly group (≥ 75 years). These comorbidities are well-known risk factors for delayed wound healing, infection, and cardiovascular events. However, in our study, these factors did not translate into increased surgical complications. This may reflect stringent perioperative medical management and careful patient selection. Nevertheless, the numbers of orthopedic events and all-cause mortality were small, with a wide confidence interval (Table 5). Thus, these results should be interpreted with caution. Despite these limitations, our findings are supported by prior work indicating that controlled comorbidities do not necessarily preclude good outcomes in minimally invasive spinal surgery. However, it is important to note that our elderly group likely represents a relatively healthy subset of the broader geriatric population, as those with severe systemic illness or cognitive impairment may not have been offered surgery. This potential selection bias must be acknowledged when interpreting our findings and underscores the need for careful preoperative assessment using tools like frailty indices and geriatric risk scoring.

Previous studies have reported postoperative outcomes in elderly patients undergoing other fusion techniques, such as transforaminal lumbar interbody fusion (TLIF) and anterior lumbar interbody fusion (ALIF). Similar to our findings, patients aged ≥ 75 years had significant improvement in clinical outcomes after TLIF 34. They had non-significantly higher rates of screw loosening and cage subsidence, and lower rate of fusion, compared with patients aged 65-74 and <65 years (n=22 in each group). Nevertheless, the elderly group had a significant longer length of hospital stay than the other two groups 34. Similar results were noted in patients underwent ALIF 35. Patients aged ≥ 75 years had significantly higher risk of postoperative complications and longer length of stay, compared with those aged <65 years. In our patients who underwent OLIF, the elderly group demonstrated significant improvements in VAS, ODI, and EQ-5D scores, with no significant difference in length of hospital stay compared with younger age groups. Another recent study 36 compared OLIF and TLIF in an elderly patient population with a mean age over 72 years. OLIF was demonstrated to have significant advantages in terms of clinical outcomes, such as shorter length of stay and greater improvement of ODI, compared with TLIF. Taken together, we suggest that OLIF could be considered an option for elderly patients with degenerative lumbar spine conditions.

When considering OLIF in elderly patients, we suggest performing preoperative assessments of bone quality (e.g., bone mineral density or VBQ scores), frailty indices, and nutritional status, all of which may influence surgical outcomes. Based on our results, elderly patients had poorer bone quality, numerically higher rates of cage subsidence and lower fusion rates, as well as non-significantly higher risks of all-cause mortality and orthopedic events compared with younger patients. Counseling patients regarding expected outcomes is an important part of preoperative assessment and should be based on findings from studies with sufficiently large sample sizes. Our results provide some evidence on this topic and should be confirmed in future studies.

Our study has several limitations. First, its retrospective design may introduce selection and information bias. Second, the sample size, particularly of the elderly cohort (≥ 75 years), was relatively small (n=25). Given the relatively small number of orthopedic events and all-cause mortality (total event number=58; Table 5), our findings should be interpreted with caution. Further studies with larger sample sizes in elderly patients are warranted to confirm our findings. Third, lack of data on preoperative frailty assessment, potential selection bias toward healthier elderly patients, single-center experience, and lack of data on patient satisfaction and return to activities of daily living. Additionally, the follow-up period was limited to six months for patient-reported outcomes, which may not capture the full extent of recovery or late complications.

## Conclusion

We demonstrated that OLIF provides meaningful improvements in pain, function, and quality of life for elderly patients with degenerative lumbar spine conditions; however, older patients exhibited smaller improvements in ODI and EQ-5D, and had worse absolute postoperative outcomes. Our findings support the use of OLIF as a viable surgical option in appropriately selected elderly patients, provided that a multidisciplinary and individualized approach to care is employed. As the aging population continues to grow, minimally invasive spinal surgery will play an increasingly critical role in managing degenerative spinal disease while preserving independence and quality of life in older adults.

## Figures and Tables

**Figure 1:**
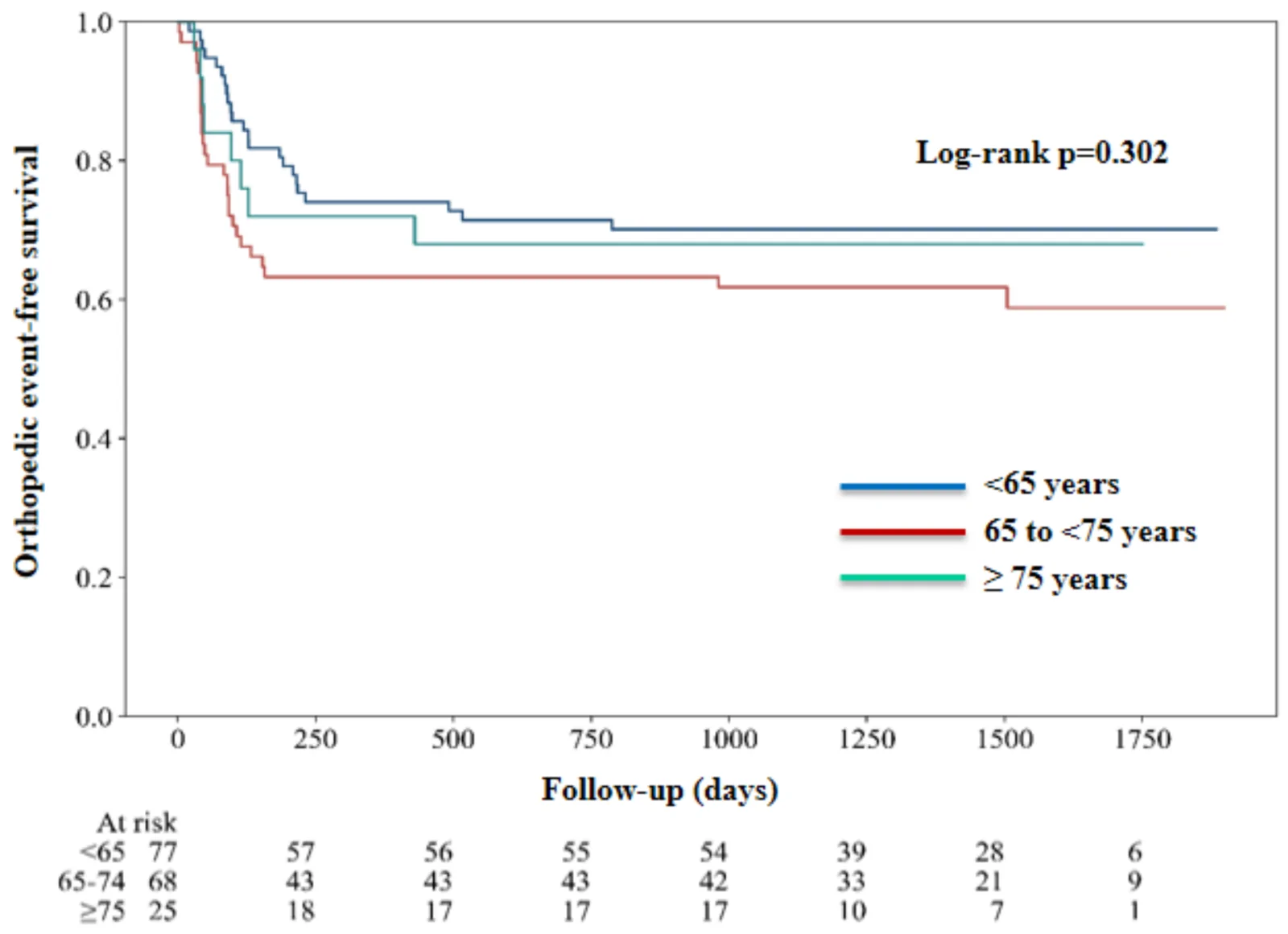
Kaplan-Meier survival curves according to age at the time of surgery (<65 years, 65 to <75 years, and ≥ 75 years). The y-axis represents orthopedic event-free survival (including screw loosening, cage subsidence, and revision surgery).

**Table 1:** Baseline characteristics of the study patients

	Patient groups by age			
Variables	< 65 years	65 to <75 years	≥ 75 years	P value
Number of patients	77	68	25	
Age, years	56.1 ± 7.3	68.9 ± 2.6	77.0 ± 2.3	<0.001
Male sex, n (%)	31 (40.3)	27 (39.7)	8 (32.0)	0.749
Body mass index, kg/m^2^	26.2 ± 3.7	25.9 ± 3.5	25.8 ± 3.5	0.879
Smoking, n (%)	12 (15.6)	3 (4.4)	4 (16.0)	0.073
Diabetes, n (%)	16 (20.8)	23 (33.8)	13 (52.0)	0.010
Hypertension, n (%)	31 (40.3)	35 (51.5)	20 (80.0)	0.003
VBQ	2.93 ± 0.53	3.19 ± 0.54	3.35 ± 0.49	0.002
VAS	8.0 ± 1.3	8.0 ± 1.5	7.8 ± 1.4	0.852
ODI	52.2 ± 8.8	57.3 ± 9.7	61.3 ± 7.1	<0.001
EQ-5D	0.37 ± 0.07	0.36 ± 0.09	0.35 ± 0.04	0.410

Values are mean ± SD or n (%). Abbreviations: EQ-5D, EuroQol-5D; ODI, Oswestry Disability Index; VAS, visual analog scale; VBQ, vertebral bone quality.

**Table 2:** Changes in patient-reported outcome measures by age

	Preoperation	At 1-month	Δ	P^a^	At 3-month	Δ	P^a^	At 6-month	Δ	P^a^
VAS										
< 65 years	8.0 ± 1.3	3.9 ± 1.9	-4.0 ± 1.9	<0.001	3.3 ± 2.2	-4.6 ± 2.2	<0.001	2.8 ± 2.4	-5.2 ± 2.3	<0.001
65 to <75 years	8.0 ± 1.5	4.1 ± 1.5	-3.9 ± 1.7	<0.001	3.4 ± 1.8	-4.7 ± 2.0	<0.001	2.9 ± 1.8	-5.1 ± 2.1	<0.001
≥ 75 years	7.8 ± 1.4	4.3 ± 1.6	-3.3 ± 1.8	<0.001	3.8 ± 1.4	-3.8 ± 1.9	<0.001	3.8 ± 2.2	-3.8 ± 2.2	<0.001
P across 3 groups	0.852	0.530	0.264		0.598	0.232		0.176	0.042	
ODI										
< 65 years	52.2 ± 8.8^bc^	43.2 ± 9.8^bc^	-8.7 ± 10.3	<0.001	33.5 ± 13.4^bc^	-18.2 ± 13.5	<0.001	25.4 ± 15.9^bc^	-26.3 ± 14.6^c^	<0.001
65 to <75 years	57.3 ± 9.7^b^	48.5 ± 8.3^bd^	-8.8 ± 10.7	<0.001	41.2 ± 10.6^bd^	-16.3 ± 11.1	<0.001	34.1 ± 12.9^bd^	-23.1 ± 12.9^d^	<0.001
≥ 75 years	61.3 ± 7.1^c^	55.6 ± 7.8^cd^	-4.9 ± 8.9	0.014	49.0 ± 5.9^cd^	-11.5 ± 7.3	<0.001	48.2 ± 7.0^cd^	-12.2 ± 9.9^cd^	<0.001
P across 3 groups	<0.001	<0.001	0.265		<0.001	0.078		<0.001	<0.001	
EQ-5D										
< 65 years	0.37 ± 0.07	0.59 ± 0.09	0.22 ± 0.11	<0.001	0.65 ± 0.13^c^	0.27 ± 0.14	<0.001	0.71 ± 0.13^bc^	0.34 ± 0.12^c^	<0.001
65 to <75 years	0.36 ± 0.09	0.57 ± 0.06	0.21 ± 0.10	<0.001	0.62 ± 0.09	0.26 ± 0.10	<0.001	0.66 ± 0.11^bd^	0.30 ± 0.14^d^	<0.001
≥ 75 years	0.35 ± 0.04	0.57 ± 0.04	0.22 ± 0.05	<0.001	0.58 ± 0.04^c^	0.23 ± 0.04	<0.001	0.57 ± 0.05^cd^	0.21 ± 0.05^cd^	<0.001
P across 3 groups	0.410	0.261	0.970		0.023	0.244		<0.001	<0.001	

Values are mean ± SD. Abbreviations: EQ-5D, EuroQol-5D; ODI, Oswestry Disability Index; VAS, visual analog scale. ^a^P value compared with preoperation. ^bcd^P<0.05 in Bonferroni post-hoc analysis for pairwise comparisons.

**Table 3:** Changes in patient-reported outcome measures by linear mixed-effects models

	VAS		ODI		EQ-5D	
	β (95% CI)	P	β (95% CI)	P	β (95% CI)	P
Time (vs Preoperation)						
At 1-month	-4.047 (-4.475, -3.619)	<0.001	-8.878 (-11.298, -6.458)	<0.001	0.219 (0.196, 0.241)	<0.001
At 3-month	-4.686 (-5.120, -4.251)	<0.001	-18.636 (-21.092, -16.180)	<0.001	0.278 (0.255, 0.301)	<0.001
At 6-month	-5.159 (-5.614, -4.704)	<0.001	-26.587 (-29.161, -24.014)	<0.001	0.337 (0.313, 0.361)	<0.001
Age group (vs < 65 years)						
65 to <75 years	0.022 (-0.567, 0.611)	0.941	5.173 (1.656, 8.689)	0.004	-0.013 (-0.042, 0.017)	0.405
≥ 75 years	-0.190 (-1.000, 0.619)	0.644	8.839 (4.004, 13.673)	<0.001	-0.018 (-0.059, 0.022)	0.374
Time x Age group interaction	---	0.234	---	<0.001	---	<0.001

Abbreviations: EQ-5D, EuroQol-5D; ODI, Oswestry Disability Index; VAS, visual analog scale.

**Table 4:** Surgical outcomes by age

	Patient groups by age			
	< 65 years	65 to <75 years	≥ 75 years	P value
Number of surgical levels	2.3 ± 1.6	2.2 ± 1.4	2.2 ± 1.5	0.882
Operation time, min	478 ± 112	454 ± 135	390 ± 143	0.012
Blood loss, ml	571 ± 552	538 ± 416	332 ± 291	0.085
Hospital stay, days	7.8 ± 4.7	7.7 ± 2.9	9.6 ± 4.4	0.128
Screw loosening, n (%)	7 (9.1)	5 (7.4)	1 (4.0)	0.702
Cage subsidence, n (%)	14 (18.2)	23 (33.8)	6 (24.0)	0.095
Revision surgery, n (%)	7 (9.1)	2 (2.9)	1 (4.0)	0.265
Fusion, n (%)	70 (90.9)	54 (79.4)	20 (80.0)	0.123

Values are mean ± SD or n (%).

**Table 5:** Risk of any orthopedic events^a^ and all-cause mortality in the study patients by age

	Unadjusted model		Multivariate adjustment^b^	
	Hazard Ratio (95% CI)	P	Hazard Ratio (95% CI)	P
Any orthopedic events^a^ or all-cause mortality				
< 65 years (event number: 23)	Reference group		Reference group	
65 to <75 years (event number: 27)	1.537 (0.881, 2.682)	0.130	1.331 (0.673, 2.636)	0.411
≥ 75 years (event number: 8)	1.153 (0.516, 2.577)	0.729	0.810 (0.291, 2.253)	0.687

^a^Screw loosening, cage subsidence, or revision surgery. ^b^Adjusted for sex, body mass index, and vertebral bone quality.

## Data Availability

Data is provided within the manuscript. The datasets used and/or analyzed during the current study are available from the corresponding author on reasonable request.

## Abbreviations

ALIF: anterior lumbar interbody fusion
EQ-5D: EuroQol-5 Dimension
ODI: Oswestry Disability Index
OLIF: oblique lateral interbody fusion
PROM: patient-reported outcome measure
TLIF: transforaminal lumbar interbody fusion
VAS: visual analog scale
VBQ: vertebral bone quality
